# Long‐term effects of a tornado: Impacts on woody native vegetation and invasive Amur honeysuckle (*Lonicera maackii*) in an urban forest

**DOI:** 10.1002/ece3.10890

**Published:** 2024-03-11

**Authors:** Theresa M. Culley, Marjorie S. Bécus, Guy N. Cameron

**Affiliations:** ^1^ Department of Biological Sciences University of Cincinnati Cincinnati Ohio USA

**Keywords:** density, disturbance, diversity, honeysuckle, invasive, *Lonicera maackii*, recruitment, tornado

## Abstract

As tornados become increasingly common with global climate change, recovery of the woody vegetation in temperate forests is imperative to maintain an intact ecosystem. In many urbanized landscapes, invasive species are also increasing and could interfere with natural recovery from environmental disturbance. We quantified the impact and 17‐year recovery from a major tornado in a temperate deciduous forest. We used vegetational surveys in southwestern Ohio at the Harris M. Benedict Nature Preserve, where approximately a third of this site was damaged by a tornado in 1999. Plots were established in the tornado‐damaged area and the nearby undisturbed forest to examine forest recovery of trees/saplings, shrubs and vines, and tree seedlings during 2003, 2006, 2010, and 2016/2017. The number of tree saplings, shrubs, and vines increased immediately after the tornado, but then declined by 2010, relative to the undisturbed forest. Forest tree recruitment was lower in tornado‐damaged sites with fewer tree seedlings, but more saplings. Tree diversity was also affected by *Agrilus planipennis* (Emerald Ash borer) which targeted native ash trees within this time period. Despite an initial increase in shrubs and vines in the damaged area, the diversity and density of shrubs approached equality in both sites by 2016. Most shrubs in both sites were the invasive *Lonicera maackii* (Amur honeysuckle). In tornado sites, honeysuckle thinned out over time, leaving larger shrubs with greater mean basal diameter compared to the undisturbed forest. Other woody invasive species were also more prevalent in the damaged area, but increased in number in both locations by 2017. The forest has the capability to begin to recover from the initial tornado, but its future composition may differ from its initial trajectory due to invasive species, loss of ash trees, and anthropogenic impacts within the urban landscape.

## Graphical abstract

Invasive Amur honeysuckle (*Lonicera maackii*) initially increased in the forest damaged by a tornado (black dotted line) compared to the undisturbed forest (solid gray line). Over 17 years, the number of honeysuckle shrubs declined, but those that remained were much larger is size (basal area), especially in damaged areas.

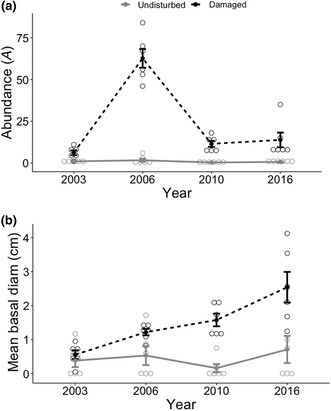

## 1 INTRODUCTION

Infrequent natural disasters such as fires, droughts, windstorms, or floods can have profound and often long‐lasting impacts upon forests (e.g., Dale et al., 2001; Goodlett, 1954). Windstorms can be powerful, but their duration may be minutes, unlike other natural disasters such as hurricanes, fires, or floods that may last from hours to weeks (Foster, 1988a, 1988b; Peterson, 2000a). Changing atmospheric convection processes associated with climate change in the United States have contributed to more extreme temperatures and more variable precipitation which may be contributing to recent increases in windstorm frequency and intensity (Berz, 1993; Karl et al., 1996; Moore, 2017). For example, the number and variability of tornadoes and supercell hurricanes increased drastically from 1995 to 2006, with further increases in number and intensity during 2012–2017 (Brooks et al., 2014; Changnon, 2009; Cody et al., 2017; Emanuel, 2017; Moore, 2017; Rahmstorf, 2017; Wurman et al., 2021). Unfortunately, urban sprawl and built environments, particularly in densely populated metropolitan areas, also have increased the risk of tornado damage (Rosencrants & Ashley, 2015).

Tornadoes are a major natural disturbance in forests of eastern North America (Peterson, 2000a) and major windthrows may even occur repeatedly within a given area (Goodlett, 1954). These windstorms can have dramatic and long‐lasting impacts on forests. They can create landscape heterogeneity, impact soils due to uprooted trees, upset landscape equilibrium by resetting succession, alter ecological processes depending on the size, shape, and configuration of the affected habitat, and reduce short‐term carbon storage (Goodlett, 1954; McNulty, 2002; Turner, 2010; Turner et al., 1998) as well as impacting animals (Barber & Widick, 2017; Hopton et al., 2009; Uetz et al., 2009) and facilitating the introduction of non‐native species (Daniels & Larson, 2020). Tornadoes also alter plant species composition by impacting the arrival and survival of propagules, and open new habitats for invasion by introduced species (but see Romano et al., 2013). These changes in turn can drive patterns of succession and affect dominance of species differentially, as not all species will be directly affected (Batista & Platt, 2003; Peterson & Rebertus, 1997; Turner et al., 1998; White et al., 2014). Fast growing dominant tree species may be more susceptible to damage than slower growing dominant species, damage from uprooting can be more important than breakage, and older stands at higher elevations may be more susceptibility to windthrow (Foster, 1988a; Peterson, 2007). This is particularly true of trees with greater diameter at breast height (DBH; Peterson, 2007; Rich et al., 2007; Walker, 1991), although damage to tree size varies among species and with windstorm intensity (Canham et al., 2001). Early successional tree species are at greater risk of windthrow in southern boreal forests, but not in midwestern forests (Peterson, 2007; Rich et al., 2007).

Although many studies have focused on the immediate effects of tornado damage, fewer have followed the long‐term recovery of these forests. What is known after a tornado is that tree resprouting can occur immediately (Glitzenstein & Harcombe, 1988; Peterson, 2000b, 2007), but a third or more of these trees may die within a few years. The size of affected trees is also important as there is a decrease in re‐sprouting and an increase in mortality with greater DBH (Peterson, 2000b, 2007). Peterson and Pickett (1995) followed regeneration in an old‐growth forest for 6 years after tornado disturbance, noting that shade‐intolerant herbs and a shade‐tolerant tree species appeared early, but herbs declined after a few years and shade‐tolerant tree species were surpassed by a tree species of intermediate tolerance. Peterson and Rebertus (1997) found that 58% of trees sprouted only 14 months after a tornado, and the understory was quickly dominated by fast‐growing, shade‐intolerant herbs.

In contrast, investigations of long‐term recovery of forests from windstorms indicate that forest composition may eventually recover to resemble pre‐disturbance vegetational composition. For example, 40 years after a windstorm initially blew down a stand of old‐growth hardwoods in Michigan, dominance by *Acer saccharum* (sugar maple) was similar to that found in undisturbed, old‐growth forests in the same region (Dahir & Lorimer, 1996). Similarly, recovery of an eastern deciduous maple‐ash‐oak forest in northern Kentucky was followed for 30 years after being struck by an F4 tornado in 1974 (Held et al., 1998, 2006; Held & Bryant, 1989; Held & Winstead, 1976). In the comparable undamaged forest, sugar maple was dominant with *Fraxinus americana* (white ash) and *Quercus* sp. (oak) being subdominant. Thirty years later, sugar maple in the damaged area was still more dominant than other tree taxa, and this continued into the understory, with sugar maple nearly excluding other tree species at the sapling stage, although white ash and oak remained as overstory subdominants. These long‐term studies illustrate the resilience of forests wherein pre‐windstorm dominant and subordinate tree species at least maintained their importance.

In addition to monitoring forest regeneration after a major natural disturbance, another important aspect of forest recovery is whether invasive species appear and persist more readily in damaged forests (Diez et al., 2012). Invasive plants are known to quickly colonize habitats damaged by hurricanes (Horvitz et al., 1998; Steiner et al., 2010), but little is known of how invasive woody plants impact long‐term natural recovery and succession of the understory in temperate forests following a tornado. Given that non‐native invasive species exhibit earlier/rapid seed germination compared to non‐invasive congeners (Gloria & Pyšek, 2017), invasive species are often more likely to establish in disturbed natural areas over time. Two years after a derecho event in a large southern Illinois national forest, very few non‐native invasive species had invaded the disturbed areas (Romano et al., 2013), but more invasives had appeared in the same area 9 years later (Daniels & Larson, 2020). Studies of vegetational recovery following a tornado are particularly absent in forests located in urban areas, in which invasive plant species may readily spread (Borden & Flory, 2021) and serve as sources of propagule dispersal. By rapidly spreading and increasing in abundance in disturbed sites, invasive plant species may alter the rate and/or direction of recovery as well as species composition of the recovered forest.

To examine long‐term recovery of an urban deciduous forest following a major tornado, we monitored tree, shrub, and vine vegetation over a 17‐year period in a tornado‐damaged area compared to a nearby undisturbed forest within the same nature preserve in southwestern Ohio. We addressed the following questions: (1) For mature trees/saplings, shrubs and vines, does species diversity, richness, abundance, and density differ between the tornado‐damaged area and the undisturbed forest, and does this change over time? (2) Is recruitment of tree species affected by tornado damage, as measured by tree seedling abundance and diversity? (3) Has invasive *Lonicera maackii* (Amur honeysuckle; hereafter honeysuckle) and other non‐native species increased in abundance in the damaged area compared to the undisturbed forest? This woody Asian shrub is now one of the most prominent invasive species in southwestern Ohio, where it is often associated with edge habitats and disturbance (Dillon et al., 2018).

## 2 METHODS

### 2.1 Study site

**FIGURE 1:**
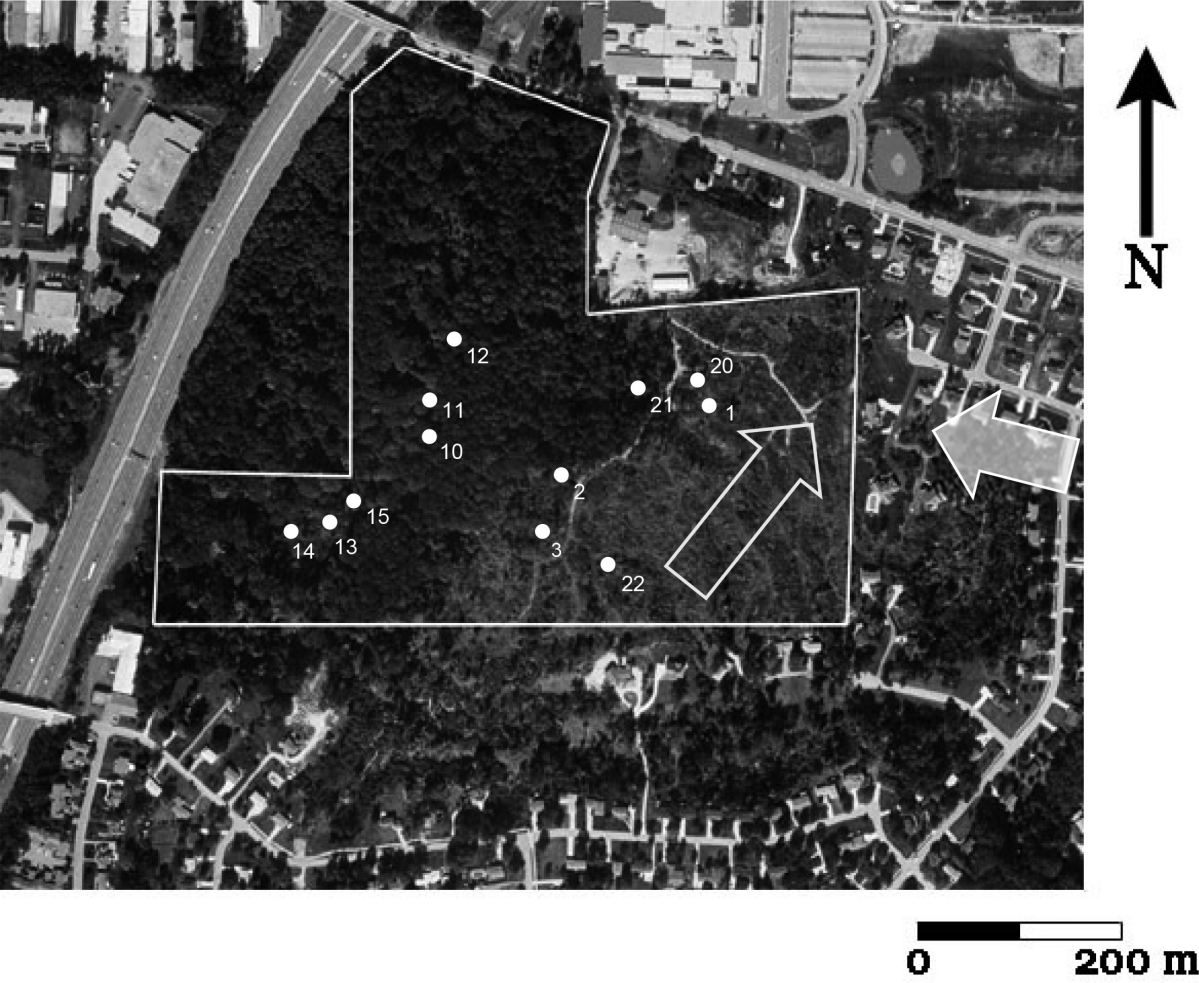
Harris Benedict Nature Preserve, Hamilton County, Ohio surrounded by houses, commercial firms, and an interstate highway. Dark area (western 2/3 of preserve) is the undisturbed forest, although some micro‐bursts took out some trees, and the lighter area (eastern 1/3 of preserve) is the area impacted by the tornado. The upper triangular‐shaped, forested parcel next to the interstate freeway is privately owned. The open arrow indicates the direction of the tornado and the opaque arrow shows direction of image in Figure 2. Permanent plots were located in the tornado‐damaged area (Plots 1–3, 20–22) and in the undisturbed forest (Plots 10–15). Photographed 23 September 2001, USGS Digital Orthophoto.

Our study used the University of Cincinnati's Harris M. Benedict Nature Preserve (formerly Hazelwood Botanical Preserve), located 26 km northeast of Cincinnati in Montgomery, Ohio (39°15′50.34″ N, 84°21′16.08″ W). This 26.2 ha preserve was established in the late 1920s in a rural portion of Hamilton County, Ohio, in a mixed mesophytic forest (Bryant, 1987; Bryant & Held, 2004), but is now an urban island bordered by residential neighborhoods, commercial properties, and an interstate highway (Figure 1). Originally, the preserve had a mature eastern deciduous forest dominated by *Fagus grandifolia* (American beech), *Acer saccharum* (sugar maple), *Quercus alba* (white oak), *Liriodendron tulipifera* (tulip poplar), *Q. rubra* (red oak), *Nyssa sylvatica* (sour gum), *Ulmus americana* (American elm), *Prunus serotina* (black cherry), and *Juglans cinerea* (bitternut hickory) with sparse forest‐floor herbaceous vegetation (Bécus, 1984; Braun, 1916; Segelken, 1929). White oak and tulip poplar were selectively logged in the 1880s, and about 12 ha of the preserve was cultivated until the early 1900s, but has since converted to second‐growth eastern deciduous forest dominated by sugar maple with some remaining patches of old‐growth forest (Bécus, 1984; Segelken, 1929). The most abundant native shrub is *Lindera benzoin* (spicebush), represented as scattered individuals. In the late 1950s, non‐native honeysuckle began to invade deciduous forests around Cincinnati (Braun, 1961), and is now widespread throughout the region and considered invasive in Ohio. Amur honeysuckle was originally planted by governmental agencies along roadsides for erosion control and was also a popular landscaping shrub (Luken & Thieret, 1996). Honeysuckle is dispersed as seed by birds and spreads quickly, forming a dense mid‐story canopy with individual shrubs exceeding 5–6 m in height (Luken & Thieret, 1996).

**FIGURE 2:**
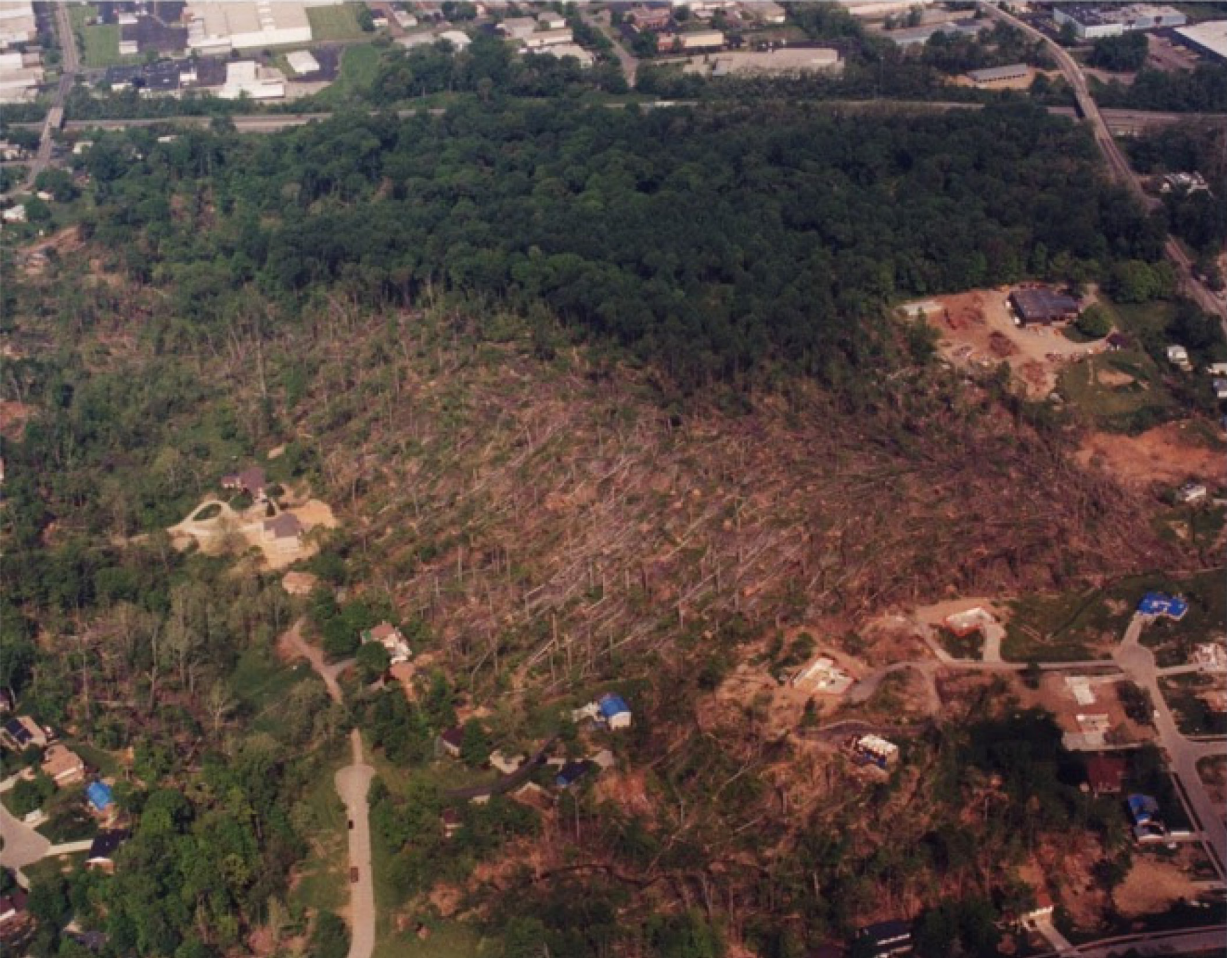
Impact of the April 9, 1999 tornado that struck the Harris M. Benedict Nature Preserve (formerly Hazelwood Preserve). Image is orientated towards the west (see Figure 1), with the I‐75 freeway along the top. Note the toppled trees in the foreground in the tornado‐disturbed area and the relatively intact forest in the background. Image obtained from Earth Explorer, Eros Data Center, U.S. Geological Survey 2002.

**FIGURE 3:**
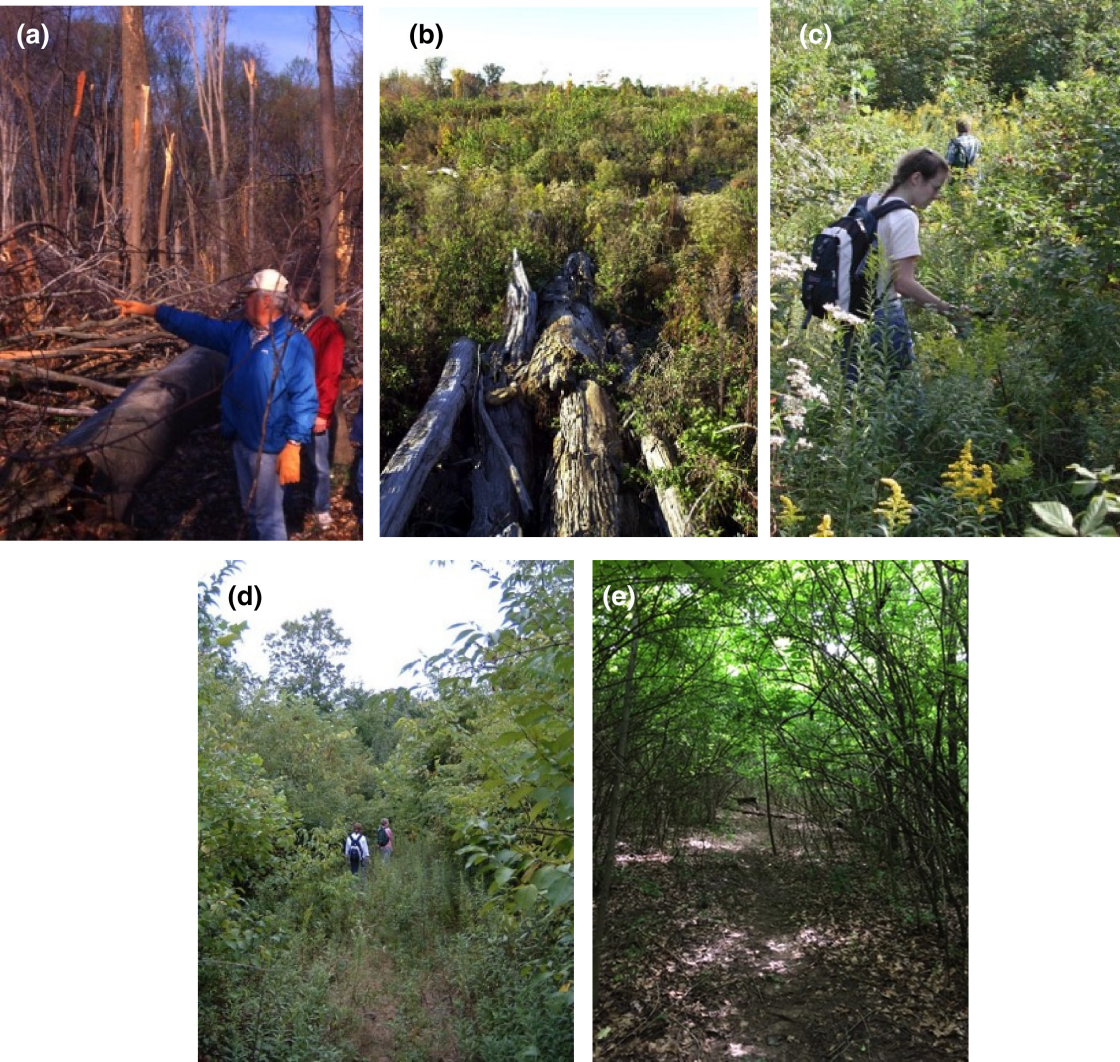
Vegetational change after the 1999 tornado at the Harris M. Benedict Nature Preserve (formerly Hazelwood Preserve). Shown are images from the tornado‐damaged area in (a) April 1999, immediately following the tornado, (b) 2001, (c) 2003, (d) 2006, and (e) 2016. All images were taken within the tornado‐damaged area although not at the same exact location. Images are taken by the authors.

**FIGURE 4:**
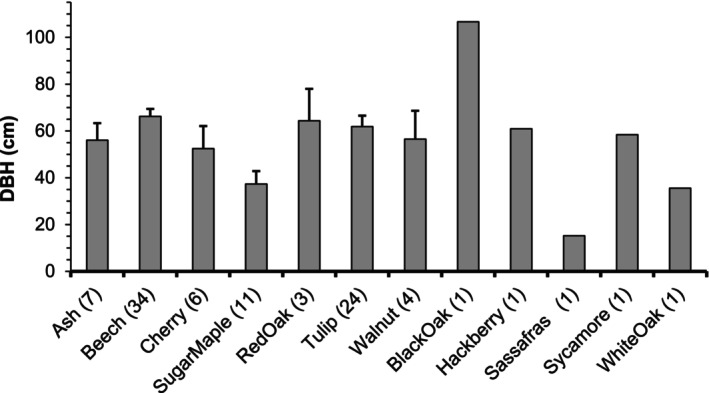
Mean (+ SE) diameter at breast height (DBH) and species of trees downed by tornado in 1999. Number of trees measured are given in parentheses. Majority of the trees were uprooted with fewer broken off above ground: ash (6 uprooted, 1 broken), beech (23, 11), cherry (6, 0), sugar maple (11, 0), red oak (1, 2), tulip poplar (20, 4), walnut (3, 1), black oak (0, 1), hackberry (1, 0), sassafras (1, 0), sycamore (0, 1), white oak (1, 0). Data courtesy of M.C. Miller and B.J. Moller.

On 9 April 1999, an F3 tornado (254–332 kph winds; Fujita 1971; EF‐3‐4, Enhanced Fujita Tornado Scale) leveled approximately 33% of the forest preserve (8.75 ha; measured from USGS Digital Orthophoto; Figures 1, 2, 3). EF‐3 to EF‐4 tornados are relatively rare in the central hardwoods region, accounting for only 0.8%–4.4% of tornados in upland hardwood forests such as the Benedict Preserve (Peterson et al., 2016). After the disturbance, downed trees at the site within the path of the tornado ranged in size from 5.1 to 106.8 cm DBH with a majority of these trees being completely uprooted rather than broken off at the trunk (Figure 4). It was impossible to hike through the damaged area due to the number of downed trees laying on their sides.

### 2.2 Vegetation censusing

We censused trees, shrubs, and vines in 2003, 2006, 2010, and 2016 (4, 7, 11, and 17 years after the tornado) in late summer and early fall (late August to early December). All woody species could be readily identified during this time period. Tree seedlings were surveyed in late summer (primarily August and September) when plants were still easily identifiable in the field; seedlings were surveyed during the same years, except the last census was in 2017 instead of 2016. We used 6 plots in the tornado‐damaged area (numbered 1–3, 20–22) and 6 plots in the nearby relatively undisturbed forest (numbered 10–15; see Figure 1). See Appendix 1 for geographic coordination points. Plots #1–3 were established relatively immediately after the tornado by J. Luken while all other plots were randomly selected at the preserve just before sampling in 2003. Most plots were positioned on relatively flat terrain, with the exception of plot #22, which was located in a ravine. Each plot was marked by a permanent 2‐m tall metal pole at its center. In the undisturbed forest, we circumscribed a circle 15‐m in diameter (sample area = 177 m^2^) around each central pole and censused all woody vegetation rooted within the plot. We then identified and counted all mature trees, tree saplings, shrubs, and vines in the plot. For mature trees and saplings (defined as over 1 m tall), we measured and recorded DBH. To quantify tree recruitment, we identified and estimated percent cover of tree seedlings (defined as less than 1 m tall) in 1 × 1‐m quadrats, each placed 2 m from the central pole in each cardinal direction within each circular plot.

Because vegetation in the tornado damaged area was very dense in 2003 (3 years after the tornado) and difficult to maneuver through, we were unable to accurately census vegetation in the same fashion as in the undisturbed forest. Instead, we laid out two 15‐m long transect lines in a north–south and east–west orientation, intersecting at 90° on the central pole in each plot. We identified and counted all mature trees, tree saplings, shrubs, and vines within 30 cm of either side of each transect line (sample area per plot = 17.64 m^2^). DBH was measured for mature trees and saplings All other surveys in the tornado‐damaged area in subsequent years were conducted as in the undisturbed areas. In each year, we also identified and counted tree seedlings in a 1 × 1 m quadrats placed 2 m from the central pole along the transect lines in each cardinal direction (sample area = 4 m^2^ per plot).

### 2.3 Data analysis

For each plot in the tornado damaged areas and undisturbed forest at each time period, we calculated mean estimates of species diversity (Shannon–Weiner *H*), species richness (*S*, number of species), abundance (*A*, number of individuals), density (*D*, number of individuals per sample area), and relative density (*RA*, not analyzed further) for three groups: (1) mature trees and saplings, (2) tree seedlings, and (3) shrubs and vines. In addition, we also included DBH for mature trees and saplings, percent cover for tree seedlings, and basal diameter for shrubs and vines. Basal area (*BA*) was also computed for each tree as Π × (DBH^2^/4), and then summed across all trees per plot within each sampling year to compute total basal area per plot. These vegetational estimates were each compared between the tornado‐damaged and undisturbed plots, using two‐way ANOVAs to examine the fixed factors of disturbance (tornado vs. undisturbed) and time (2003, 2006, 2010, and 2016/2017). All raw data were normally distributed except for the following: *S* was log‐transformed for Trees and Shrubs/Vines; *D* was arcsin‐square‐root transformed for Trees but log‐transformed for Shrubs/Vines; *A* was arcsin‐square‐root transformed for Trees and log‐transformed for Shrubs/Vines; *BA* was square‐root transformed for trees; and Mean DBH (Trees), Mean Diameter (Shrubs/Vines), and Percent Cover (Seedlings) were each log‐transformed. We also examined abundance, density, and basal diameter of honeysuckle. Because *A* and basal diameter data of this shrub were non‐normally distributed even following various transformations, a Kruskal–Wallis test was used to compare honeysuckle in plots in the tornado‐damaged and undisturbed sites within each year.

## 3 RESULTS

### 3.1 Mature trees and saplings

Immediately following the tornado in 1999, 94 large trees had been toppled (uprooted) in the southern portion of the preserve (Figures 1 and 2). Most (74%) of these trees consisted of dominant species at the site (Figure 4): American beech (36%), tulip poplar (26%), and sugar maple (12%). Other species included black cherry, ash, oaks, *Juglans nigra* (walnut), *Sassafras albidium* (sassafras), *Celtis occidentalis* (hackberry), and *Plantanus occidentalis* (sycamore).

**FIGURE 5:**
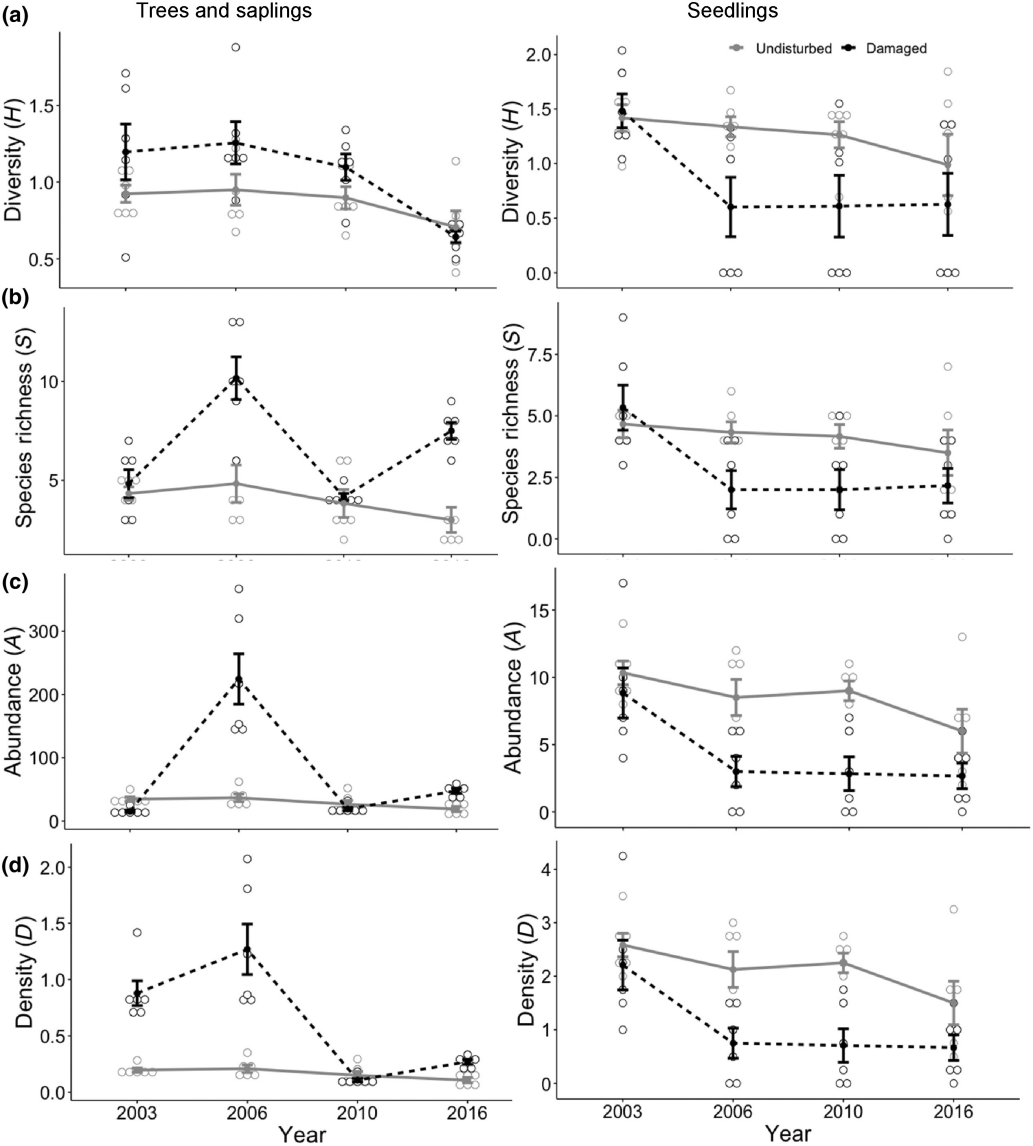
Response of mature trees and saplings (left) and tree seedlings (right) in the tornado damaged area (dotted black lines) and the undisturbed forest (gray solid lines). Shown are the (a) Shannon Weiner diversity index – *H*, (b) species richness – *S*, (c) abundance – *A*, and (d) density – *D*. The tornado occurred in April 1999.

**FIGURE 6:**
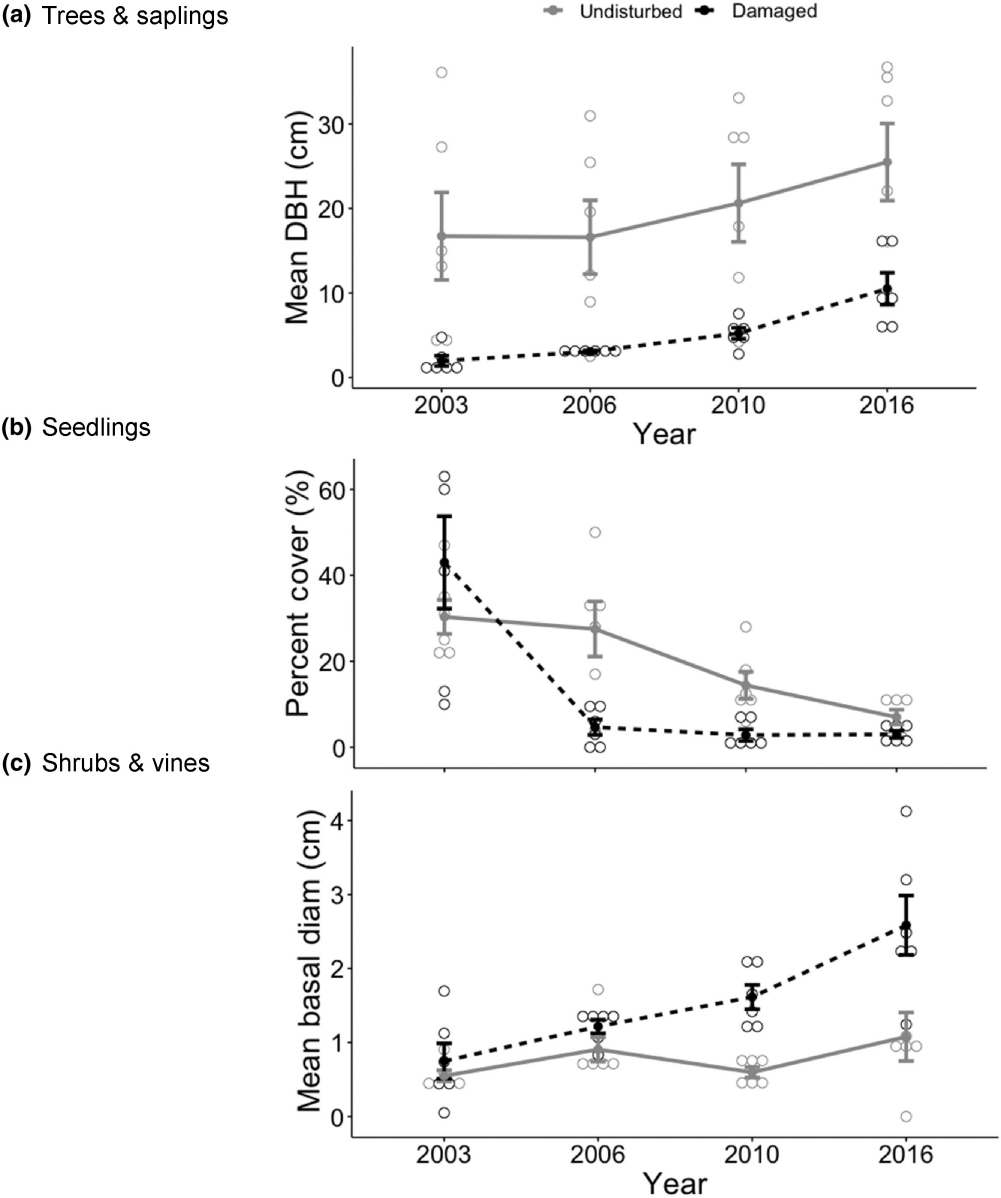
Measures of plant size or cover through time in the forest damaged by a tornado (black dotted line) and the undisturbed forest (solid gray line). Shown are (a) mean DBH (cm) of mature trees and saplings, (b) percent cover of tree seedlings in 1 m^2^ plots, and (c) mean basal diameter (cm) of shrubs and vines. The tornado occurred in April 1999.

**FIGURE 7:**
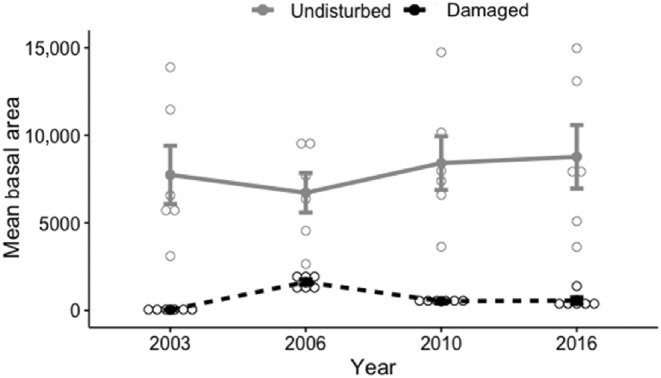
Basal Area for trees in plots within the tornado‐damaged area and in the undisturbed area, based on individual tree DBH. This follows Peterson (2000a, 2000b), who noted that measures of windthrow effects on tree abundance may differ depending on whether abundance is measured as tree density/plot (including distances among trees) or as tree basal area (including only tree DBH). Shown are cumulative data across all plots per location and per year.

Species diversity of mature trees and saplings was higher in the tornado‐damaged areas (1.05) than in undisturbed forest (0.87; *F*_[1,40]_ = 5.67, *p* = .022) within each sampling period, except for 2016, resulting in a difference across sampling periods (*F*_[3,40]_ = 6.66, *p* < .001; Table 1; Figure 5a). Species richness of mature trees and saplings were significantly higher in the tornado‐damaged sites (6.67) compared to the undisturbed forest (4.00; *F*_[1,40]_ = 28.99, *p* < .0001), reflecting a significant increase in species in the damaged area in 2006 and 2016 (*F*_[3,40]_ = 6.49, *p* = .001; Figure 5b). As expected after a tornado, abundance of trees and saplings was lower in the damaged forest in 2003, but peaked in 2006, resulting in higher abundance in the damaged forest (76.7) compared to the undisturbed forest (29.2), although not significantly different across years due to large variation in the data (*F*_[1,40]_ = 0.401, *p* = .531; Figure 5c). Abundance was similarly low in both sites for 2006 and 2016. Density per plot was overall higher in the damaged area (0.631) than in the undisturbed forest (0.165) within the first two sampling periods only (*F*_[1,40]_ = 92.98, *p* < .0001; Figure 5d). Mean DBH of trees and saplings remained consistently lower in the tornado‐damaged sites (mean = 5.20 cm) compared to the undisturbed areas (19.86 cm; *F*_[1,40]_ = 55.823, *p* < .0001), even though DBH consistently increased across all years in both locations (*F*_[3,40]_ = 7.23, *p* < .001; Figure 6a). Total tree basal area was substantially lower in the damaged sites (689.56 cm^2^) than in the undamaged forest (7912.41 cm^2^; *F*_[1,40]_ = 200.23, *p* < .0001; Figure 7) where several dominant trees were quite large (over 50 DBH). Basal area also tended to increase over time in the intact forest, as expected (*F*_[3,40]_ = 1.88, *p* = .027).

**TABLE 1:** Average species diversity/plot (Shannon–Weiner *H*′), average species richness/plot (*S*), abundance (*A*), density/plot (*D*), and relative density (*RD*) for mature trees and saplings, seedlings, and shrubs and vines in the tornado‐damaged forest and undisturbed forest in each year.

Year	Plot	Trees and saplings					Tree seedlings					Shrubs and vines				
		*H*′	*S*	*A*	*D*	*RD*	*H*′	*S*	*A*	*D*	*RD*	*H*′	*S*	*A*	*D*	*RD*
2003	Damaged	1.20	4.83	15.5	0.88	0.14	1.48	5.33	8.8	2.21	0.08	0.73	2.83	8.3	0.47	0.15
	Undisturbed	0.92	4.33	34.7	0.20	0.03	1.42	4.67	10.3	2.58	0.09	0.57	2.17	11.0	0.06	0.02
2006	Damaged	1.26	10.17	224.5	1.27	0.14	0.60	2.00	3.0	0.75	0.04	0.64	4.50	79.3	0.45	0.15
	Undisturbed	0.95	4.83	36.8	0.21	0.02	1.34	4.33	8.5	2.12	0.12	0.16	1.33	9.2	0.05	0.02
2010	Damaged	1.10	4.17	19.2	0.11	0.07	0.61	2.00	2.8	0.71	0.04	0.20	1.50	12.3	0.07	0.12
	Undisturbed	0.90	3.83	26.5	0.15	0.10	1.26	4.17	9.0	2.25	0.13	0.33	1.67	5.0	0.03	0.05
2016/2017	Damaged	0.64	7.50	47.7	0.27	0.12	0.63	2.17	2.7	0.67	0.05	0.30	2.00	16.0	0.09	0.13
	Undisturbed	0.71	3.00	18.8	0.11	0.06	0.99	3.50	6.0	1.50	0.11	0.27	1.33	4.2	0.02	0.03

**FIGURE 8:**
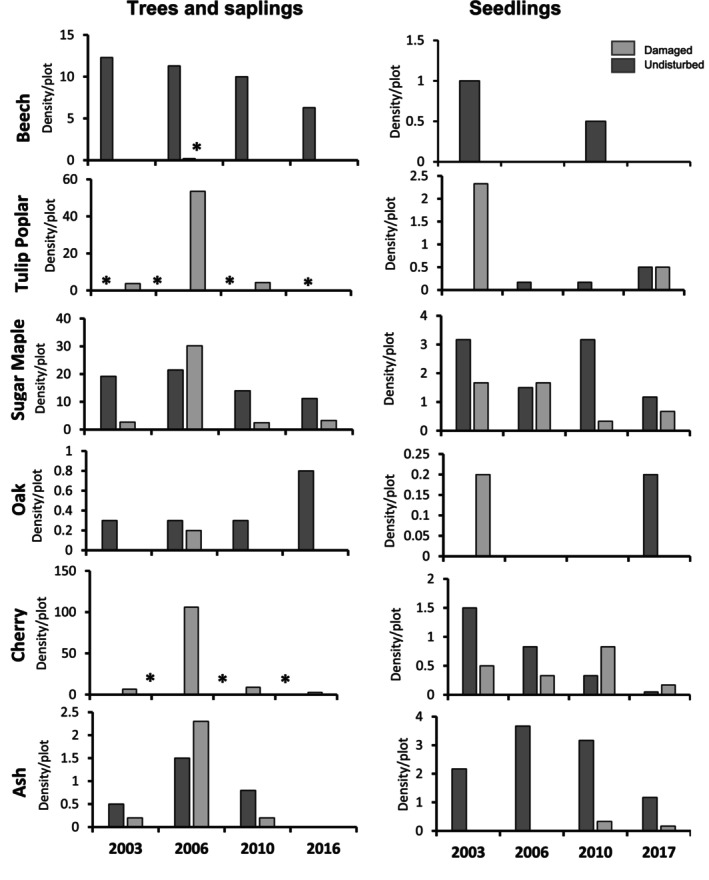
Density of the five most common tree species observed in the tornado‐damaged site (light gray bars) and the undisturbed forest (dark gray bars) for both trees and saplings (left column) and seedlings (right column) across the four sampling periods. An asterisk indicates a non‐zero value for that treatment and year.

Six native tree species and their seedlings were the most abundant in our study (Figure 8): American beech, red oak, *Quercus velutina* (black oak), black cherry, sugar maple, tulip poplar, and *Fraxinus americana* (white ash). However, white ash was already undergoing substantial decline in the area following attack by the non‐native insect borer. *Pyrus calleryana* (Callery pear) was the only non‐native tree species observed in the study, recorded as only a few saplings in the tornado‐damaged area in 2003, 2006, and 2016 (Appendix 2). Data on density/plot, relative density, and DBH of all trees and seedlings in the damaged areas and undisturbed forest are provided in Appendices 2 and 3.

Beech was the most common tree species downed by the tornado (Figure 8, Appendix 2), and did not reappear in the damaged site, except for scattered saplings in 2006 (density = 0.2/plot, see * in Figure 8). In contrast, beech trees in the undisturbed forest remained at relatively high densities throughout all sampling periods. Tulip poplar was the second‐most common tree destroyed by the tornado, but it only occurred as large scattered individuals in the undisturbed forest (0.2–0.5/plot, see * in Figure 8). After the tornado, tulip poplar saplings appeared in the damaged sites, including a temporary surge in 2006 of 53.5 saplings/plot. Sugar maple was the third most common large tree species prior to the tornado (Figure 8, Appendix 2), usually found within the undisturbed forest. Sugar maple saplings persisted in all sampling periods in the damaged site, with the largest number in 2006 (30.2 saplings/plot). Oak species were already relatively rare in the undisturbed forest and previously in the tornado‐damaged area (Figure 8), only appearing in the damaged sites in 2006. Consistent with early successional species, cherry was not abundant in the established forest prior to the tornado (Figure 8) and had low density in the undisturbed forest during all years (0.2–1.0/plot, see * in Figure 8). After the tornado however, cherry saplings were consistently found in the damaged plots, with a temporary surge in 2006 of 106 saplings/plot. Finally, density of white ash was similar in both sites. By 2016, ash had disappeared from both areas, as it did throughout southwestern Ohio.

### 3.2 Tree seedlings

Species diversity of tree seedlings was typically lower in the tornado‐damaged sites (0.83) than in the undisturbed forest (1.25; *F*_[1,40]_ = 4.56, *p* = .039; Table 1) except directly after the tornado in 2003, when species diversity values were similar (Figure 5e). Similarly, species richness of tree seedlings was significantly lower overall in the damaged sites (2.88) than in the undisturbed forest (4.17; *F*_[1,40]_ = 6.40, *p* = .016), with richness declining over time in the tornado damaged sites (*F*_[3,40]_ = 3.81, *p* = .017; Figure 5f). There was a lower abundance of tree seedlings in the damaged sites (4.33) compared to the undisturbed forest (8.46; *F*_[1,40]_ = 21.06, *p* < .001), with similar values only in 2003 directly after the tornado (Figure 5g). Density/plot of tree seedlings was significantly lower in the damaged forest (1.08) than in the undisturbed forest (2.11; *F*_[1,40]_ = 21.06, *p* < .0001) in all years except in 2003 (Figure 5h). Total percent cover of tree seedlings was initially highest in the damaged sites but then substantially decreased 3 years later, where seedling percent cover was much lower than in the undisturbed forest (*F*_[3,40]_ = 12.88, *p* < .0001). This resulted in an overall significant difference in percent cover of seedlings in the tornado damaged area (53.5%) compared to the undisturbed forest (79.25%; *F*_[1,40]_ = 20.80, *p* < .0001; Figure 6b).

Density of seedlings of different tree species varied across years and forest types. Beech tree seedlings were completely absent in the damaged area across all years, and were only inconsistently found in the undisturbed forest in 2003 and 2010 (Figure 8). Density of tulip poplar seedlings in the tornado damaged site were highest in 2003 before disappearing for the subsequent two sampling periods and resurfacing in 2017. In the undisturbed forest, tulip poplar seedlings were absent in 2003, but then increased slowly in density over the next three sampling periods. Sugar maple seedlings were found in all sampling periods in both forest types, but generally were more often found in the undisturbed forest. Oak seedlings were sparse overall, only occurring in 2003 in the damaged forest and 2017 in the undisturbed forest. In contrast, cherry tree seedlings occurred in both damaged and undisturbed sites within each sampling year, but were most abundant in the undisturbed forest in 2003 and 2006, and remained at the damaged sites for the remaining two sampling periods. In the damaged forest, ash seedlings were absent during 2003–2006, and only very sparse in 2010–2017. In contrast, density of white ash seedlings in the undisturbed forest was relatively much higher in all years, although they did decline over time. In both cases, ash seedlings most likely originated from an extant seed bank, as any extant adult trees died over time due to borer damage. Data on density/plot, relative density, and percent cover of tree seedlings in the damaged and undisturbed forest areas are provided in Appendix 3.

### 3.3 Shrubs and vines

**FIGURE 9:**
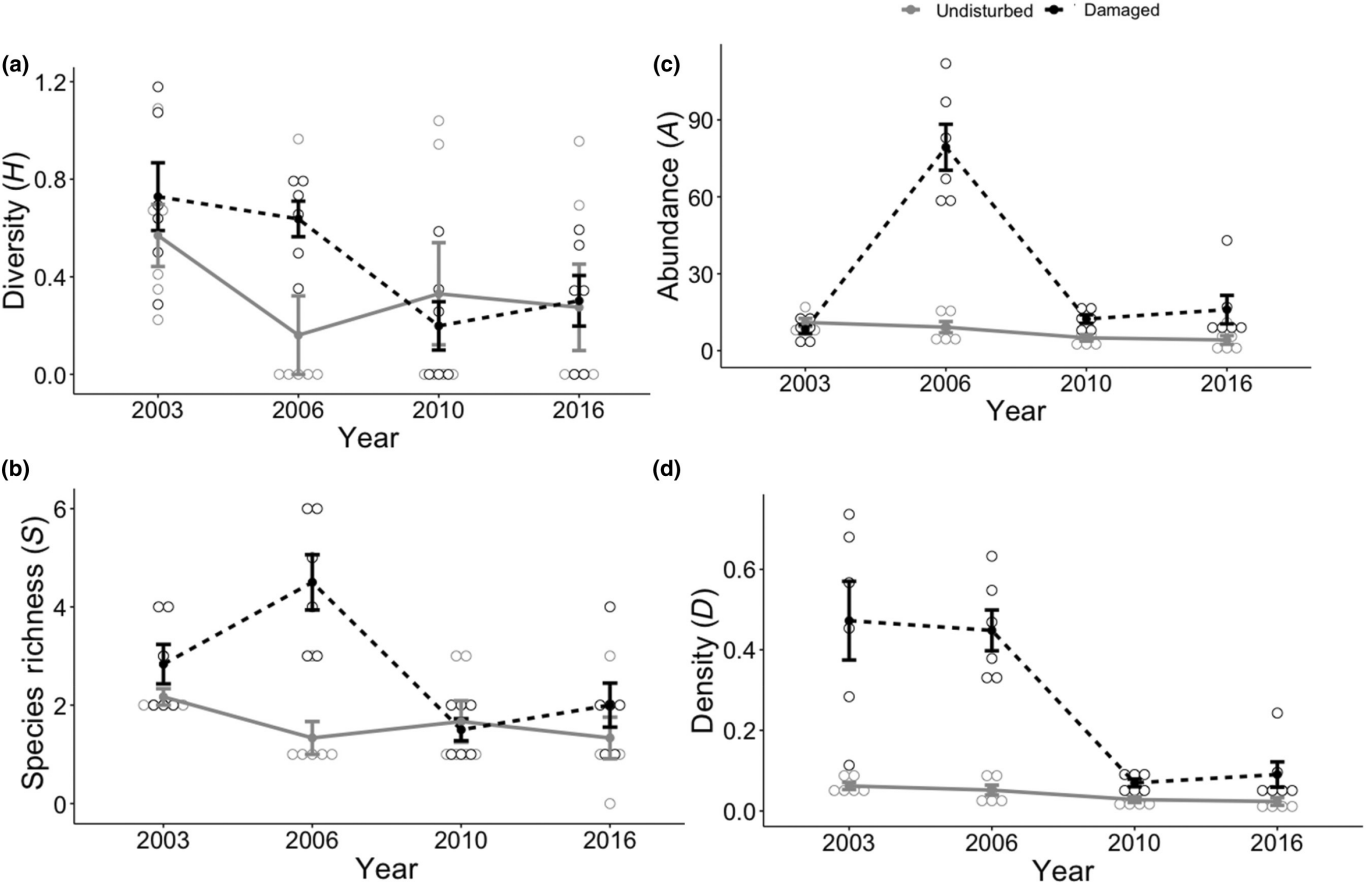
Responses of shrubs and vines in the tornado damaged area (black dotted lines) and the undisturbed forest (gray solid lines). Shown are (a) the Shannon–Weiner diversity index – *H*, (b) species richness – *S*, (c) abundance – *A*, and (d) density – *D*. The tornado occurred in April 1999.

Species diversity of shrubs and vines was similar overall in damaged areas (0.467) and undisturbed forest (0.334; *F*_[1,40]_ = 1.73, *p* = .196), despite diversity being highest in 2006 in the tornado damaged areas (Table 1; Figure 9a). Species richness was overall higher in the damaged sites (2.71) compared to the undisturbed forest (1.62; *F*_[1,40]_ = 12.959, *p* = .001), and especially so in 2006 (Figure 9b). Abundance of shrubs and vines was also significantly higher in the tornado damaged sites (29.0) than in the undisturbed forest (7.33; *F*_[1,40]_ = 35.21, *p* < .0001), although values initially did not differ in 2003 (Figure 9c). Shrub/vine density per plot was much higher in the damaged forest (0.271) than the undisturbed forest (0.041; *F*_[1,40]_ = 83.28, *p* < .0001), with the highest diversity recorded in the damaged area in 2003 and 2006 (Figure 9d). Overall, shrubs also were significantly larger in the tornado‐damaged sites (mean basal diameter = 5.86 cm) compared to the undisturbed forest (3.13 cm; *F*_[1,40]_ = 20.92, *p* = <0.0001) with this larger size difference increasing over time (*F*_[1340]_ = 7.48, *p* < .001; Figure 6c).

The most abundant shrubs and vines in our study were introduced honeysuckle, followed by *Smilax hispida* (native greenbrier), *Viburnum acertifolium* (maple leaf viburnum), and *Lindera benzoin*. Species with lower abundance included native species of *Rubus* (blackberry/raspberry), *Vitis riparia* (grape), and *Rhus radicans* (poison ivy), as well as non‐native *Lonicera japonica* (Japanese honeysuckle), *Ligustrum* (privet), and *Rosa multiflora* (multiflora rose). Density/plot and mean stem diameter of shrubs and vines sampled in the damaged and undisturbed forest areas are available in Appendix 4.

**FIGURE 10:**
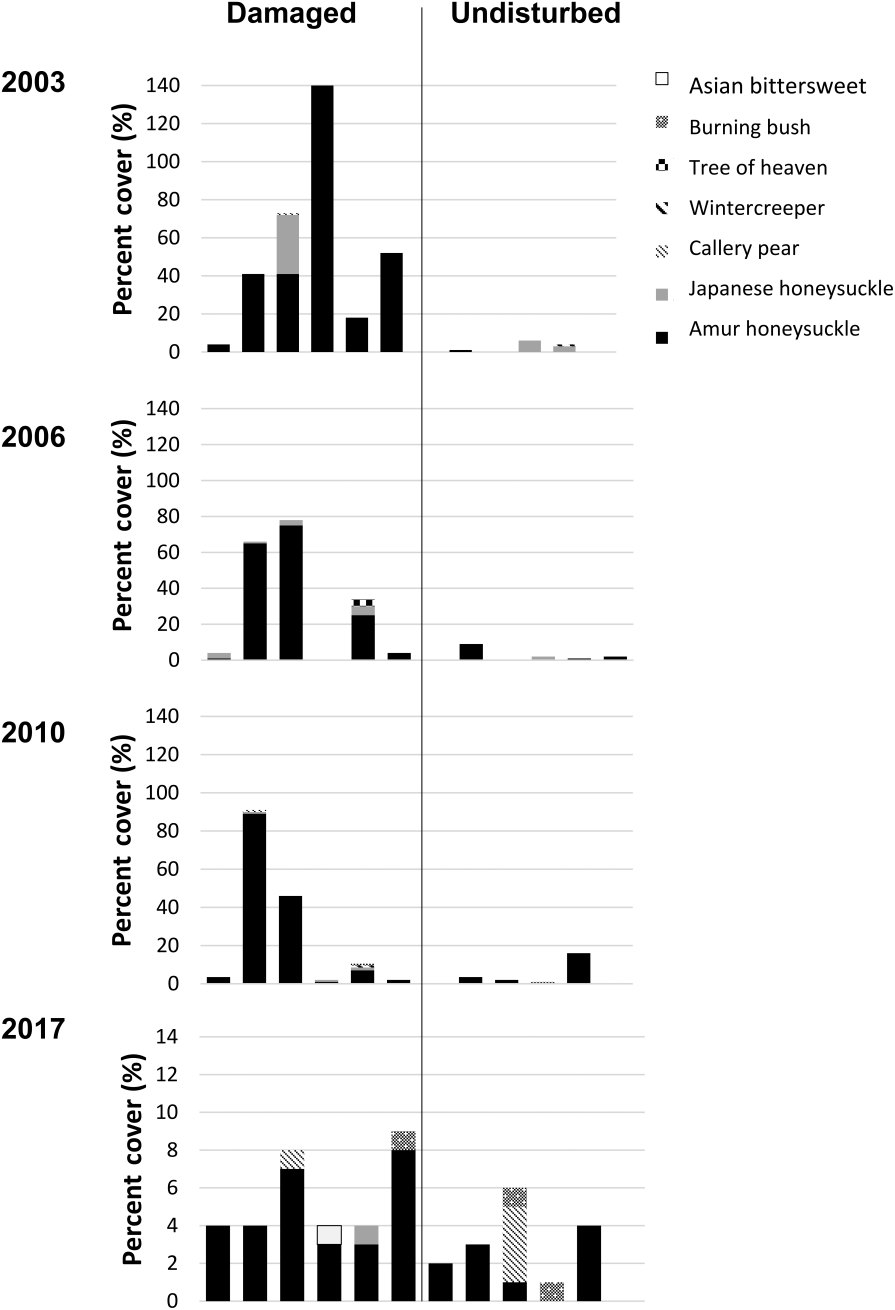
Percent cover of non‐native, woody species classified as invasive within each of the six tornado‐damaged and the six undisturbed plots (on the *x*‐axis) within each year of sampling. Note that the *y*‐axis is the same for all years except 2017.

**FIGURE 11:**
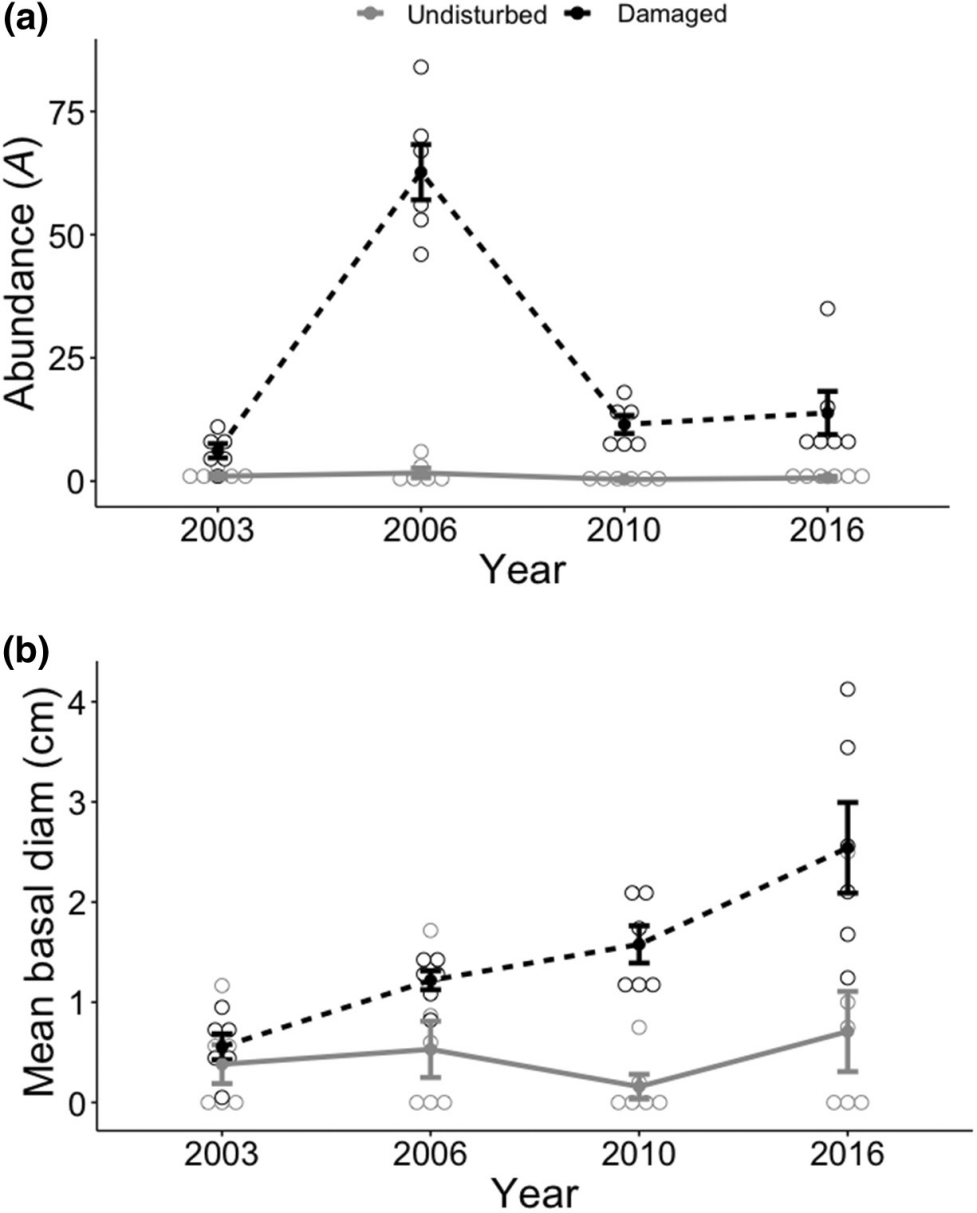
Performance of invasive Amur honeysuckle (*Lonicera maackii*) through time in the forest damaged by a tornado (black dotted line) and the undisturbed forest (solid gray line). Shown are (a) abundance, and (b) mean basal diameter (cm). The tornado occurred in April 1999. Note that (a) and (b) are nearly identical to Figures 9c and 6c respectively, because honeysuckle makes up the majority of shrubs at the site.

In general, invasive woody species tended to occur more often in the tornado‐damaged sites than in undamaged areas, as evident by percent cover in the plots (Figure 10). This pattern was consistent across the first three sampling periods. Percent cover of invasive woody shrubs and vines was still relatively low in 2017, but there was a greater variety of invasive species in both damaged and undisturbed plots (Figure 10). Invasive honeysuckle exhibited the highest density in the damaged area, especially in 2006 (Figure 10), while honeysuckle density was very low (0.2–0.8/plot) before ultimately disappearing within plots in the undisturbed forest. Overall, there was significantly higher abundance of honeysuckle in the damaged forest compared to the undisturbed forest within each year sampled: 2003 (Kruskal‐Wallis $X_{\left[1\right]}^{2}$ = 6.29, *p* = .012), 2006 ($X_{\left[1\right]}^{2}$ = 8.42, *p* = .004), 2010 ($X_{\left[1\right]}^{2}$ = 8.67, *p* = .003), and 2016 ($X_{\left[1\right]}^{2}$ = 8.49, *p* = .004; Figure 11). Individual honeysuckle shrubs were similar in size in both sites in 2003 ($X_{\left[1\right]}^{2}$ = 0.936, *p* = .333) and 2006 ($X_{\left[1\right]}^{2}$ = 3.15, *p* = .076), but shrubs in the tornado damaged area exhibited greater basal diameter in 2010 ($X_{\left[1\right]}^{2}$ = 8.61, *p* = .003) and 2016 ($X_{\left[1\right]}^{2}$ = 5.85, *p* = .016; Figure 11). Both greenbrier and maple leaf viburnum exhibited similar patterns of absence (Appendix 4): in the undisturbed forest, both species exhibited the greatest density in 2003 immediately after the tornado, before declining in abundance in 2010–2016 (greenbrier was absent in 2006 and maple leaf viburnum exhibited its lowest density in 2016). In contrast, density of spicebush in the damaged sites was initially very low in 2003, before peaking in 2006, and then disappearing. Within the undisturbed forest, spicebush density followed a similar pattern with a peak in 2006, but remained low in abundance.

## 4 DISCUSSION

Even though major environmental disturbances such as tornados are now increasing in frequency due to global climate change (Moore, 2017; Peterson, 2000a), the long‐term response of vegetation remains relatively understudied (but see Daniels & Larson, 2020; Held et al., 2006). In this 17‐year study of a beech‐maple forest in southwestern Ohio, an F3 tornado had long‐lasting impacts on the vegetational composition and continues today to shape the forest's regeneration. Even after 17 years, woody plant abundance, species diversity and richness still differed between the undisturbed and tornado‐damaged plots. This was true of mature/sapling trees, tree seedlings, and shrubs and vines, and especially of Amur honeysuckle and other invading species. At Benedict Preserve, it is unlikely that the damaged forest will be able to regain the same vegetational composition it once had, in part due to the regional extirpation of ash and invasion by non‐native plants from the surrounding urban area. In addition, seedling recruitment now almost exclusively favors sugar maple. Prevalence of sugar maple was also detected in northern Kentucky 30 years after a tornado (Held et al., 1998, 2006; Held & Bryant, 1989; Held & Winstead, 1976; Jones‐Held et al., 2019). Other woody species such as oak and tulip poplar at the Benedict Preserve will likely persist in small numbers within the damaged area, given the proximity to seed‐producing adult trees nearby in the undisturbed forest. In other studies, disturbance selected strongly against the leading dominant tree species (Glitzenstein & Harcombe, 1988), although expectations for long‐term recovery were variable. The unknown factor at Benedict Preserve is the impact of invasive honeysuckle, which can negatively impact forest regeneration by reducing growth and survival of native tree seedlings of sugar maple and red oak (Gorchov & Trisel, 2003), resulting in lower seedling density and species richness (Hutchinson & Vankat, 1997).

As predicted following a major disturbance, diversity, richness, density per plot, and abundance of trees, saplings, shrubs, and vines all increased within the first 7 years following the tornado, before returning to similar rates as in the undisturbed forest. This initial pulse in 2006 of increased plant abundance and sapling density across all tree species occurred in light and space gaps created by the downing of large trees by the tornado. Interestingly, this pulse was not detected in new seedlings after the tornado, suggesting that only older seedlings and saplings persisted through the tornado event while conditions may have not been not conducive for seed germination and/or survival of the seed bank. Sapling abundance and density per plot declined after this initial pulse, likely following self‐thinning due to shading as the forest canopy reformed and loss of ash to Emerald Ash Borer (Herms & McCullough, 2014); a drought year in 2010 further decreased sapling survivorship.

This initial vegetational pulse followed by a slight to moderate decline in abundance or species richness has also been detected in other vegetational studies of tornado impacts. Increased woody species richness was seen for 7 years after a tornado struck a northwestern Pennsylvania forest (Peterson & Pickett, 1995). This included higher tree seedling density and percent cover in disturbed plots compared to more intact areas. In our study, seedling density and percent cover was significantly lower across all years in tornado‐damaged sites, compared to the adjoining undisturbed forest. This difference was not likely due to the wind event, which was severe in both cases – an F4 tornado in Peterson and Pickett (1995) and an F3 tornado in the current study. Rather it may be a result of the type of forest, which was a very large national forest used by Peterson and Pickett (1995), and a much smaller forest remnant surrounded by residential and commercial areas in our study. At Benedict Preserve, any recruitment would be limited to the pre‐tornado seed bank and from seed dispersal from the remaining trees in the intact forest portion of the much smaller preserve.

We also found dramatic differences in species composition and abundance of mature trees and saplings between the tornado‐damaged area and the undisturbed forest, with implications for long‐term response of the forest. Variation in abundance of tree species in the undisturbed forest likely reflects the presence of new saplings and die off of existing saplings. For example, increased species diversity in later sampling periods in the undisturbed forest reflected only few individuals of subdominant species such as bitternut hickory, *Nyssa sylvatica* (black gum), *Acer negundo* (box elder), *Cornus* sp. (dogwood), *Carpinus caroliniana* (hornbeam), and *Asimina triloba* (pawpaw). Abundance of beech trees naturally declined over time while abundance of sugar maple and ash fluctuated. As an early successional species, black cherry was relatively rare in the undisturbed forest, with its modest density reflecting saplings and small trees. Similarly, tulip poplar also exhibited low abundance in all years, likely due to its shade intolerance.

As Peterson (2007) noted, values of tree abundance or density (which accounts for the number of individuals and the spacing between them) are often much higher than values of basal area (i.e., based only on the trees themselves). This was also seen in the plots we examined (Figure 5), where many saplings were notably abundant following the tornado, as they grew in open gaps left by the toppled trees. These saplings self‐thinned over time, eventually resulting in lower basal area with fewer plants surviving to the final sampling period; those that did survive were only of moderate size. In contrast, trees and saplings in the undisturbed forest were fewer in number (i.e., lower abundance and density per plot) but persisted over time, with much larger trees (over 50 cm DBH) contributing to the larger basal area of the undamaged plots.

The tornado also dramatically altered the composition of shrubs and vines at the Benedict Preserve. In the tornado‐damaged area, native green brier and maple leaf viburnum disappeared, eventually followed by spicebush, whereas all three native species persisted in the undisturbed forest nearby. These three native species are shade‐adapted and may not have been able to survive in the high light opened up by loss of the forest canopy. The only exception was invasive honeysuckle, which was consistently very rare in the undisturbed forest, but quickly gained a foothold in damaged plots, increasing dramatically in abundance by 2006. In Ohio, honeysuckle multiplies rapidly along forest edges in high light (Dillon et al., 2018) and also has an extended growing season, being one of the first shrubs to leaf out in early spring and one of the last to drop its leaves in late fall (Luken & Thieret, 1996). Over time at the Benedict Preserve, the number of honeysuckle shrubs declined and naturally self‐thinned, leaving behind much larger individuals with greater basal diameter, in comparison to the undisturbed site. A similar initial pulse of percent cover of honeysuckle was also observed approximately 8 years following tornados in southern Illinois (Daniels & Larson, 2020), likely due to preference of the species for high light environments.

### 4.1 Tree recruitment

As measured in plots at the Benedict Preserve, tree recruitment was also negatively impacted by the tornado. After 7 years, there were fewer seedlings overall in the damaged area, with significant differences in abundance and diversity of seedlings between tornado‐damaged areas and undisturbed forest. The only exception was sugar maple, already a dominant species with many reproductive individuals in the nearby intact forest. Sugar maple seedlings were found growing in both damaged and undisturbed sites across all sampling periods; consequently, sugar maple still comprises the majority of the recruitment cohort in the preserve. But in the damaged plots, there was no recruitment of beech, formally a dominant tree before the tornado, even though beech seedlings continued to appear in the undisturbed forest nearby. In contrast, Peterson and Pickett (1995) reported that seedling density of beech remained high and consistent 6 years after a tornado struck a Pennsylvania maple‐hemlock forest. In our study, another dominant species, tulip poplar, produced a dense number of seedlings in the damaged area in 2003, which could explain the very large number of tulip poplar saplings in 2006 (54 per plot); unfortunately, tulip poplar seedlings had all but disappeared in following years in the damaged site but remained in the nearby undisturbed forest. Give that tulip poplar is shade intolerant, it was likely outcompeted by the dense vegetation during the pulse year. Other tree species once prevalent prior to the tornado, such as ash and oak, disappeared or were very rare in the damaged area by the end of the study, although they could still be found scattered in the nearby forest. In fact, many of the largest trees downed by the tornado were oaks (Figure 4) in which greater diameter is associated with higher mortality (Shirakura et al., 2006). Black cherry seedlings were observed in both damaged and undisturbed plots in all years, but larger trees and saplings were only detected in the damaged site (peaking in 2006 with 106 per plot), as would be expected for an early successional species. Overall, tree recruitment in areas damaged by the tornado is expected to continue, but with greater emphasis on sugar maple and tulip poplar from the existing seed bank. The seed bank will eventually be exhausted unless replenished by seed from the nearby undisturbed forest (especially for beech, which was completely eradicated by the tornado).

These results clearly reflect differences in abundance and species composition between the undisturbed and the tornado‐damaged forest over time. Species diversity and richness of mature trees and saplings over all years were higher in the tornado‐damaged forest than in the undisturbed forest. The trend was reversed for tree seedlings, in which species diversity and species richness were higher in the undisturbed forest than in the tornado‐damaged forest. It is possible that the newly opened light gaps in the damaged area and later the intense shade offered by the invasive honeysuckle inhibited the germination and growth of woody seedlings. If so, this could be detrimental for long‐term response of the Benedict Preserve forest, especially as honeysuckle shrubs naturally thin out, but become much larger in size and canopy coverage over time. Overall, these results indicate that the subsequent forest will have a different vegetational composition and appearance than what it was prior to the tornado. The composition will likely switch from beech‐tulip poplar‐maple‐ash to primarily only sugar maple with some tulip trees, considering what saplings and seedlings are persisting today in the damaged area.

### 4.2 Impact of woody invasive species

Large‐scale disturbances such as windstorms are often associated with increased invasion risk (Daniels & Larson, 2020; Diez et al., 2012; Romano et al., 2013) and this study was no exception. Uprooted trees can disrupt the soil and dramatically change the microenvironment, opening up new opportunities for colonization by seeds readily dispersed via birds and wind. At Benedict Preserve, invasive honeysuckle was often found in plots within the damaged area, sometimes approaching percent cover of 140% (as bushes grew on top of one another in particularly dense thickets). In contrast, honeysuckle was rare to absent in most undisturbed plots. Due to its greater competitive ability, dispersal of seeds by birds, past historical use for erosion control along roadways, and presence as ornamental shrubs in the surrounding residential area, honeysuckle is already established in southwestern Ohio (Luken & Thieret, 1996) and can easily invade forest edges and move into interiors. In a northern Kentucky forest, honeysuckle had become a dominant component of the understory 40 years after the site was impacted by a tornado (Jones‐Held et al., 2019). However Romano et al. (2013) only saw Amur honeysuckle, *Lonicera japonica* (Japanese honeysuckle) and *Rosa multiflora* (multiflora rose) in a few plots 2 years after a derecho in southern Illinois, but Daniels and Larson (2020) noted that invasive species may increase over time but will eventually be shaded out as the forest overstory recovers. In our study, *Lonicera japonica* (Japanese honeysuckle vine) and *Ailanthus altissima* (tree of heaven) occasionally appeared in the damaged plots 7–11 years after the tornado, but many more invasive species were present by the last sampling period in 2017. These included ornamental taxa planted in the surrounding residential area such as the Callery pear, *Euonymus alatus* (winged burning bush), and *E. fortunei* (wintercreeper; Figure 11). Other well‐known invasive species were also observed, such as *Celastrus orbiculatus* (Asian bittersweet). Some of these same species were found in the undisturbed area as well, indicating that even these locations are not impervious to invasion within this urban nature preserve. With few exceptions, the invasive species detected in our study were also observed in a tornado‐damaged area in southern Illinois (Daniels & Larson, 2020). Only Callery pear and winged burning bush were not observed in the Daniels and Larson (2020) study, which may reflect the location of their study – extending across a national forest, state park, and national wildlife refuge – and not adjoining residential areas where these ornamental species are planted. Increased plant invasion in southwestern Ohio region may also be exacerbated by the death of ash trees (Herms & McCullough, 2014), which once comprised 1 in 10 trees in the state (Herms, 2007), opening new light gaps in which invasive species often thrive.

The high abundance of honeysuckle in the damaged plots in our study may reduce the abundance of native plant species, as invasive honeysuckle can negatively affect native tree species (e.g., Gorchov & Trisel, 2003; Hutchinson & Vankat, 1997). In fact, species diversity and species richness of native shrubs and vines in the undamaged forest decreased from 2003 to 2016, as the number of different woody invasives had increased in both areas by 2017. Similarly, tree seedlings exhibited declining low values of species diversity, abundance, richness, and density in the tornado damaged forest in 2010–2016 as honeysuckle became the dominant shrub. A possible explanation is that several of the woody invasives could have reduced the recruitment and survival of these native species with which they compete for resources.

As invasive species are often found after a large‐scale disturbance opens up the forest canopy, it is not surprising that honeysuckle and other woody invasive species have increased over time in tornado‐damaged plots. Glitzenstein and Harcombe (1988) reported that the *Triadica sebifera* (Chinese tallow tree) appeared in abundance as saplings a few years after a tornado‐damaged site in Texas, predicting that this invasive species may be a concern in the near future. Amur honeysuckle also increased in percent cover several years after a tornado in Illinois (Daniels & Larson, 2020) and was identified as a dominant understory species in Kentucky albeit 40 years after the tornado (Jones‐Held et al., 2019).

### 4.3 Long‐term implications

How a forest will respond to the increasing numbers of windstorm events in North America will ultimately be shaped by its geographic size and local anthropogenic pressure. Although vegetational composition of the Benedict Preserve has shifted 17 years after the tornado, other studies in Kentucky (Held et al., 2006) and Michigan (Dahir & Lorimer, 1996) reported forest community composition returned back to pre‐tornado levels 30 and 40 years later. Unlike these studies at larger and more remote locations, the Benedict Preserve is a relatively small preserve of 26.2 ha nestled within a highly urbanized matrix, surrounded by residential and commercial areas, a local high school, and bordered on one side by a major interstate freeway. In comparison, Dinsmore Woods State Nature Preserve, is a 43.3 ha site in northern Kentucky (Held et al., 1998, 2006; Held & Bryant, 1989; Held & Winstead, 1976; Jones‐Held et al., 2019), surrounded largely by undeveloped forest considered to be fairly undisturbed since the 1830's (Jones‐Held et al., 2019). Damage from the 1974 tornado at Dinsmore Woods was also patchy across the preserve, in contrast to Benedict Preserve where a swath of 33% of the forest was directly impacted. Although forest community composition was able to fully recover at Dinsmore Woods, such recovery is unlikely at Benedict Preserve, due to the smaller size of the preserve, the more severe pattern of destruction, and ongoing ecological effects of habitat fragmentation (e.g., limited seed and pollen dispersal, more edge effects, greater whitetail deer herbivory (Gorchov et al., 2021), lower rates of insect pollination) as well as propagule pressure of nearby invasive plant species and ornamentals planted nearby. Consequently, the vegetational impacts of tornadic activity at Benedict Preserve cannot be buffered by surrounding native vegetation, as might occur at larger and non‐urbanized locations like Dinsmore Woods. However, time will only tell if Benedict Preserve can recover its original vegetational composition, but the trajectory thus far indicates that it is on a different course, largely shaped by its environment within the urban landscape in which natural processes have been anthropogenically altered.

## AUTHOR CONTRIBUTIONS

**Theresa M. Culley:** Conceptualization (supporting); data curation (lead); formal analysis (lead); methodology (equal); visualization (lead); writing – original draft (equal); writing – review and editing (lead). **Marjorie S. Bécus:** Conceptualization (supporting); methodology (equal); writing – review and editing (supporting). **Guy N. Cameron:** Conceptualization (lead); formal analysis (equal); methodology (lead); writing – original draft (equal); writing – review and editing (supporting).

## FUNDING INFORMATION

Funding was provided by the Department of Biological Sciences, University of Cincinnati.

## CONFLICT OF INTEREST STATEMENT

The authors declare no conflicts of interest.

## Data Availability

Summary data for woody trees and saplings, shrubs, and vines for each of the sampled years are available in the Appendices 1, 2, 3, 4.

## APPENDIX 1

### Locations of the long‐term sampling plots within the tornado‐damaged area and the relatively undisturbed area within the Harris M. Benedict Nature Preserve, owned by the University of Cincinnati

**Table:**

Plot	Area type	Latitude	Longitude	Elevation (m)
1	Tornado	N 39.26266	W 084.35309	264
2	Tornado	N 39.26248	W 084.35360	267
3	Tornado	N 39.26209	W 084.35398	266
20	Tornado	N 39.26274	W 084.35322	266
21	Tornado	N 39.26278	W 084.35352	268
22	Tornado	N 39.26172	W 084.35382	257
10	Undisturbed	N 39.26316	W 084.35558	261
11	Undisturbed	N 39.26335	W 084.35544	262
12	Undisturbed	N 39.26393	W 084.35506	265
13	Undisturbed	N 39.26315	W 084.35609	260
14	Undisturbed	N 39.26300	W 084.35643	260
15	Undisturbed	N 39.26329	W 084.35597	263

On Nov. 25, 2023, the center of all permanent plots were still marked in the field with a metal stake. Measurements were taken with the WGS 84 map datum.

## APPENDIX 2

### Density/plot (*D*), relative density (*RD*), and mean diameter at breast height (*DBH*, cm) of mature trees and tree saplings in the disturbed tornado‐damaged areas and in undisturbed areas in 2003, 2006, 2010, and 2016. Also shown is whether the species was observed as seedlings after the tornado and is represented in the seedling dataset

**Table:**

Common name	Species name	Present as seedlings	2003			2006			2010			2016		
			*D*	*RD*	*DBH*	*D*	*RD*	*DBH*	*D*	*RD*	*DBH*	*D*	*RD*	*DBH*
Damaged														
Boxelder	*Acer negundo*	X	1.0	0.065	0.4	8.5	0.038	1.6	—	—	—	0.8	0.102	2.6
Sugar maple	*Acer saccharum*	X	2.7	0.172	0.8	30.2	0.136	3.4	2.5	0.132	4.1	3.3	0.408	7.3
Ohio buckeye	*Aesculus glabra*		0.2	0.011	0.6	—	—	—	—	—	—	—	—	—
Pawpaw	*Asimina triloba*	X	0.2	0.011	0.1	5.7	0.025	0.4		0.035	0.6	—	—	—
American hornbeam	*Carpinus caroliniana*		—	—	—	0.7	0.003	0.4	—	—	—	—	—	—
Bitternut hickory	*Carya cordiformis*	X	0.3	0.022	0.1	1.5	0.007	1.1	—	—	—	0.2	0.020	0.3
Catalpa	*Catalpa speciosa*		—	—	—	0.2	0.001	1.2	—	—	—	0.2	0.020	1.1
Hackberry	*Celtis occidentalis*	X	—	—	—	0.3	0.001	2.3	—	—	—	—	—	—
American beech	*Fagus grandifolia*		—	—	—	0.2	0.001	0.3	—	—	—	—	—	—
White ash	*Fraxinus americana*	X	0.2	0.011	0.4	2.3	0.010	2.9	0.2	0.009	0.1	—	—	—
Black walnut	*Juglans nigra*		—	—	0.2	0.001	0.6	—	—	—	—	—	—	—
Tulip poplar	*Liriodendron tulipifera*	X	3.7	0.237	1.8	53.5	0.240	3.4	4.3	0.228	3.7	—	—	—
American Sycamore	*Platanus occidentalis*		0.2	0.011	0.3	0.3	0.001	0.2	—	—	—	—	—	—
Black cherry	*Prunus serotina*	X	6.5	0.419	1.1	106.0	0.476	2.9	8.8	0.465	4.0	2.8	0.347	5.8
Callery pear	*Pyrus calleryana*	X	0.2	0.011	0.1	0.7	0.003	0.8	—	—	—	0.2	0.020	0.9
Staghorn sumac	*Rhus typhina*		0.3	0.022	1.6	1.2	0.005	4.0	0.3	0.018	4.2	0.2	0.020	2.3
White oak	*Quercus alba*	X	—	—	—	0.2	0.001	0.2	—	—	—	—	—	—
Sassafrass	*Sassafrass albidum*	X	—	—	—	9.5	0.043	1.9	1.0	0.053	3.5	0.3	0.041	3.9
Elm	*Ulmus americana*, *U. rubra*	X	0.2	0.011	0.3	1.2	0.005	1.6	1.3	0.070	1.4	0.2	0.020	0.7
Undisturbed														
Boxelder	*Acer negundo*	X	0.7	0.019	0.1	0.2	0.004	1.7	—	—	—	—	—	—
Sugar maple	*Acer saccharum*	X	19.2	0.550	4.2	21.5	0.578	4.3	14.0	0.528	6.8	11.2	0.558	10.7
Pawpaw	*Asimina triloba*		—	—	—	0.3	0.009	0.1	—	—	—	—	—	—
American hornbeam	*Carpinus caroliniana*	X	0.2	0.010	0.6	0.2	0.004	0.7	0.2	0.006	0.8	0.2	0.008	0.9
Bitternut hickory	*Carya cordiformis*	X	0.2	0.005	0.2	0.2	0.004	0.1						
Dogwood	*Cornus* sp.		0.7	0.019	1.5	0.5	0.013	1.7	0.3	0.013	0.6	—	—	—
American beech	*Fagus grandifolia*	X	12.3	0.354	8.1	11.3	0.305	6.2	10.0	0.377	11.4	6.3	0.317	16.5
White ash	*Fraxinus americana*	X	0.5	0.014	7.0	1.5	0.040	5.7	0.8	0.031	7.1	—	—	—
Tulip poplar	*Liriodendron tulipifera*	X	0.2	0.010	11.3	0.3	0.009	23.2	0.5	0.019	12.9	0.3	0.017	26.7
Black gum	*Nyssa sylvatica*	X	—	—	—	0.2	0.004	0.27	—	—	—	—	—	—
Black cherry	*Prunus serotina*	X	0.5	0.014	0.2	0.5	0.013	2.1	0.2	0.006	0.1	1.0	0.050	0.2
White oak	*Quercus alba*		0.2	0.010	8.7	0.2	0.004	8.2	0.2	0.006	9.1	0.2	0.008	9.3
Red/black oak	*Quercus rubra*, *Q. velutina*	X	0.3	0.010	19.5	0.3	0.004	10.1	0.3	0.013	21.1	0.8	0.042	13.6

## APPENDIX 3

### Density/plot (*D*), relative density (*RD*), and percent cover (*Cover*) of tree seedlings in the disturbed tornado‐damaged areas and in undisturbed areas in 2003, 2006, 2010, and 2017. Also shown is whether each species was also present as a tree or sapling.

**Table:**

Common name	Species name	Present as trees/saplings	2003			2006			2010			2017		
			*D*	*RD*	*Cover*	*D*	*RD*	*Cover*	*D*	*RD*	*Cover*	*D*	*RD*	*Cover*
Damaged														
Box elder	*Acer negundo*	X	0.17	0.02	0.33	—	—	—	—	—	—	0.17	0.06	0.17
Red maple	*Acer rubra*		0.33	0.04	0.67	—	—	—	—	—	—	—	—	—
Sugar maple	*Acer saccharum*	X	1.67	0.21	2.78	1.67	0.50	1.08	0.33	0.12	0.17	0.67	0.25	0.50
Tree‐of‐heaven	*Ailanthus altissima*		—	—	—	0.17	0.05	0.67	—	—	—	—	—	—
Pawpaw	*Asimina triloba*	X	—	—	—	—	—	—	0.17	0.06	0.33			
Bitternut hickory	*Carya cordiformis*	X	0.50	0.06	0.83	0.17	0.05	0.17	—	—	—	—	—	—
Hackberry	*Celtis occidentalis*	X	—	—	—	—	—	—	0.33	0.12	0.25	—	—	—
White ash	*Fraxinus americana*	X	—	—	—	—	—	—	0.33	0.12	0.33	0.17	0.06	0.17
Tulip poplar	*Liriodendron tulipifera*	X	2.33	0.30	6.65	—	—	—	—	—	—	0.50	0.19	0.33
Black gum	*Nyssa sylvatica*	X	—	—	0.17	0.05	0.17	—	—	—	—	—	—	—
Black cherry	*Prunus serotina*	X	0.50	0.06	0.67	0.33	0.10	0.33	0.83	0.29	0.50	0.17	0.06	0.17
Callery pear	*Pyrus calleryana*	X	0.33	0.04	0.33	—	—	—	0.33	0.12	4.67	0.17	0.06	0.17
White oak	*Quercus alba*	X	0.17	0.02	0.17	—	—	—	—	—	—	—	—	—
Red oak	*Quercus rubra*		0.17	0.02	0.50	—	—	—	—	—	—	—	—	—
Oak	*Quercus* spp.	X	—	—	—	—	—	—	—	—	—	0.17	0.06	0.17
Sassafrass	*Sassafrass albidum*	X	1.67	0.21	3.67	0.33	0.10	0.17	0.17	0.06	0.17	—	—	—
Elm	*Ulmus americana*, *U. rubra*	X	—	—	—	0.5	0.15	0.33	0.33	0.12	0.33	0.67	0.25	0.50
Undisturbed														
Box elder	*Acer negundo*	X	1.83	0.19	2.14	0.50	0.07	0.50	0.33	0.04	0.50	—	—	—
Red maple	*Acer rubra*		—	—	—	0.17	0.02	0.17	—	—	—	—	—	—
Sugar maple	*Acer saccharum*	X	3.17	0.32	3.61	1.50	0.20	1.06	3.17	0.37	1.05	1.17	0.33	0.92
American hornbeam	*Carpinus caroliniana*	X	—	—	—	0.17	0.02	0.17	—	—	—	—	—	—
Bitternut hickory	*Carya cordiformis*	X	—	—	—	0.33	0.04	0.17	0.17	0.02	0.17	—	—	—
American beech	*Fagus grandiflora*	X	1.00	0.10	0.67	—	—	—	0.50	0.06	0.33	—	—	—
White ash	*Fraxinus americana*	X	2.17	0.22	5.86	3.67	0.49	0.17	3.17	0.37	2.54	1.17	0.33	1.00
Tulip poplar	*Liriodendron tulipifera*	X	—	—	—	0.17	0.02	0.17	0.17	0.02	0.17	0.50	0.14	0.50
Black gum	*Nyssa sylvatica*	X	—	—	—	0.17	0.02	0.33	—	—	—	—	—	—
Black cherry	*Prunus serotina*	X	1.50	0.15	0.83	0.83	0.11	0.50	0.33	0.04	0.25	0.05	0.01	0.50
Callery pear	*Pyrus calleryana*		—	—	—	—	—	—	—	—	—	0.01	0.00	0.17
Elm	*Ulmus americana*, *U. rubra*		0.17	0.02	0.17	—	—	—	0.67	0.08	0.54	0.50	0.14	0.50
Black oak	*Quercus velutina*	X	—	—	—	—	—	—	—	—	—	0.17	0.05	0.17
Sassafrass	*Sassafrass albidum*		—	—	—	—	—	—	0.17	0.02	0.17	—	—	—

## APPENDIX 4

### Density/plot (*D*), relative density (*RD*), and mean stem diameter (*Stem*) of shrubs and vines in the disturbed tornado‐damaged areas and in undisturbed areas in 2003, 2006, 2010, and 2016

**Table:**

Common name	Scientific name	2003			2006			2010			2016		
		*D*	*RD*	*Stem*	*D*	*RD*	*Stem*	*D*	*RD*	*Stem*	*D*	*RD*	*Stem*
Damaged													
Privet	*Ligustrum* spp.	—	—	—	0.50	0.01	0.17	—	—	—	—	—	—
Spicebush	*Lindera benzoin*	0.5	0.01	0.43	8.00	0.10	0.82	—	—	—	—	—	—
Japanese honeysuckle	*Lonicera japonica*	—	—	—	0.50	0.01	0.25	—	—	—	—	—	—
Amur honeysuckle	*Lonicera maackii*	37.00	0.94	3.34	62.50	0.79	1.20	13.83	0.94	1.53	8.33	0.91	2.45
Muitiflora rose	*Rosa multiflora*	0.17	—	—	1.17	0.01	1.16	—	—	—	—	—	—
Raspberry	*Rubus ideaeus*	0.50	0.01	(1.0)	—	—	—	—	—	—	—	—	—
Blackberry	*Rubus* spp.	1.00	0.03	(11.0)	1.33	0.02	0.58	0.58	—	—	—	—	—
Grape	*Vitis* spp.	—	—	—	4.67	0.06	0.86	0.83	0.06	0.71	0.83	0.09	2.07
Undisturbed													
Spicebush	*Lindera benzoin*	8.17	0.75	0.61	10.83	0.76	0.80	3.83	0.77	0.56	3.17	0.75	0.64
Amur honeysuckle	*Lonicera maackii*	0.83	0.08	0.51	2.67	0.19	0.90	0.17	0.03	0.13	0.50	0.12	0.58
Greenbrier	*Smilax* spp.	0.67	0.06	0.05	—	—	—	0.17	0.03	0.13	0.22	0.05	0.22
Poison ivy	*Toxicodendron radicans*	—	—	—	—	—	—	0.17	0.03	0.25	—	—	—
Maple leaf viburnum	*Viburnum acerifolium*	1.17	0.11	0.19	0.67	0.05	0.10	0.67	0.13	0.06	0.33	0.08	0.21

In the tornado area in 2003, stem diameter was too difficult to measure for blackberries and raspberries, and so average stem count is provided instead (in parentheses).
